# Influence of plasma, surface, and angle on interlinked X-ray emission dynamics in femtosecond burst pulse ablation

**DOI:** 10.1038/s41598-025-34221-x

**Published:** 2026-01-08

**Authors:** Daniel Metzner, Philipp Rebentrost, Peter Lickschat, Thomas Lampke, Steffen Weißmantel

**Affiliations:** 1 University of Applied Sciences Mittweida, Laserinstitut Hochschule Mittweida, Mittweida, 09648 Germany; 2 Chemnitz University of Technology, Department of Materials and Surface Engineering, Chemnitz, 09125 Germany

**Keywords:** Condensed-matter physics, Plasma physics, Ultrafast lasers

## Abstract

The ablation of solid materials using ultrashort laser pulses at high intensities leads to the emission of X-rays. This effect is particularly pronounced when burst pulses are applied due to pulse-to-pulse interactions within a burst. Simultaneously, the resulting surface topography changes depending on whether single pulses or burst pulses are used. This study experimentally investigates the spectral X-ray emission during the ablation of 304L-steel with single and burst pulses, varying the detection angle and predefined laser parameters. The aim is to analyze how surface topography, which evolves during ablation, influences the measurements of X-ray emission during area irradiation. The results indicate that surface topography-induced shielding of X-ray emission occurs for single and MHz-burst pulses, but only at fluences where characteristic surface structures form. In the MHz-burst regime, additional shielding effects arise from interactions with the ablation plume, which also contribute to a shift toward higher-energy X-ray photons. In contrast, GHz-burst pulses preserve a smooth surface across all investigated fluences, preventing shielding of X-rays.

## Introduction

Ultrashort pulse laser ablation has become a widely used technique in materials processing, enabling precise micromachining with minimal thermal damage. The interaction of high-intensity femtosecond laser pulses with matter inherently leads to the emission of X-rays due to bremsstrahlung and characteristic X-ray fluorescence^1–3^. The generation of X-rays^4–6^ and X-ray dosimetry^7–13^ in laser ablation processes has been investigated in several studies, particularly concerning the influence of laser parameters such as pulse energy, repetition rate, and burst mode operation. Although it has been well established that X-ray emission intensifies at laser intensities exceeding 10$^{13}$ W/cm$^2$, recent studies^7,14–16^ have demonstrated that the introduction of pre-pulses or burst pulses can significantly enhance X-ray generation by modifying plasma formation and laser-matter coupling^12,17,18^.

One of the most recent investigations focused on GHz repetitive burst laser irradiation^19^, demonstrating that the use of GHz-double pulses with an asymmetric energy distribution (10:90 ratio) at a fixed detection angle of 10$^{\circ }$ relative to the material surface led to a drastic increase in X-ray emission compared to single pulses, attributed to the intense interaction of subsequent burst pulses with an existing laser-induced plasma. In addition, a rotating target was used, and the rotation speed was chosen to ensure geometric separation of consecutive pulses. As a result, prior pulses had no influence on surface morphology evolution, making the findings independent of topographical effects. Furthermore, the study did not consider MHz-burst pulses. However, such a regime was expected to lead to increased X-ray emission due to interactions with an existing ablation plume^12^. Complementary to these findings, the study by Rimkus et al. demonstrated a compact high-flux X-ray source from solid targets using MHz/GHz burst irradiation^20^. Operating a GHz/MHz burst amplifier at 100 kHz and 90 W average power in ambient air, they reported hard-X-ray fluxes exceeding 10$^{11}$ photons s$^{-1}$ in the 6–40 keV range. The work employed a fixed detection geometry and emphasized source performance rather than angle-resolved, morphology-coupled analysis.

The angular dependence of X-ray emission in ultrashort pulsed laser ablation has been investigated in prior research. Legall et al.^7^ examined X-ray emission at detection angles ranging from 10$^{\circ }$ to 38$^{\circ }$ relative to the sample plane and identified a maximum dose rate at approximately 29$^{\circ }$ for steel targets. Their study, which employed a fixed laser incidence and a high-repetition-rate femtosecond laser, showed that the detected X-ray dose is influenced by both material properties and laser intensity. However, while their findings provide valuable insight into angular dependencies, they did not explore the influence of surface morphology or the effects of different burst regimes on the emission characteristics.

It has been documented that ablation of steel using ultrashort single pulses and fluences well above the ablation threshold leads to the formation of cone-like protrusions^21–24^ (CLPs), while MHz-burst pulses can induce significant melt formation due to high intra-burst heat accumulation^25–30^, resulting in a morphology dominated by melt dynamics and re-solidified structures. In contrast, GHz burst pulses exhibit a pronounced surface smoothing effect, with the resulting morphology governed primarily by thermomechanical effects due to intense pulse-plasma interactions^26,31,32^ or recoil pressure^33^. This highlights the importance of investigating how laser-induced topographical changes influence X-ray emission across different detection angles and burst regimes.

This study aims to systematically investigate the angular dependence detection of X-ray emission during the ablation of 304L-steel using single, MHz- and GHz-burst double pulses. A key distinction of this study is the equal energy distribution across the pulses within a burst, allowing for a direct comparison between individual burst pulses and single pulses at comparable fluence per pulse and total burst fluence. The study is designed to mimic an application-oriented milling process, where different laser pulse regimes result in distinct surface morphologies. By systematically analyzing the X-ray emission as a function of detection angle and correlating it with the resulting surface morphology, this study provides deeper insights into the interplay between laser-induced plasma dynamics and material modification processes. The findings contribute to both fundamental understanding and practical considerations for high-power ultrashort pulse laser applications.

## Results

### Single pulse ablation

**Fig. 1:**
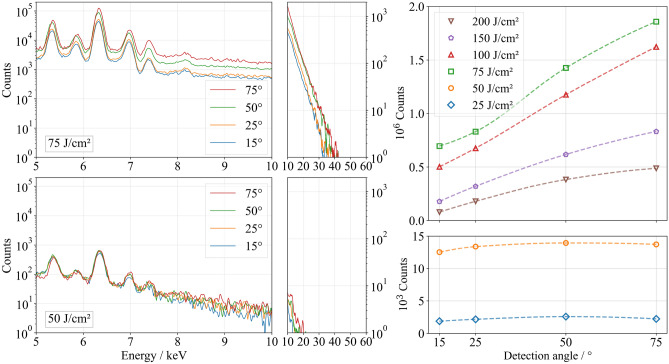
X-ray emission spectra (left) and the number of photons measured (right) as a function of the detection angle and the fluence using the single-pulse regime.

**Fig. 2:**
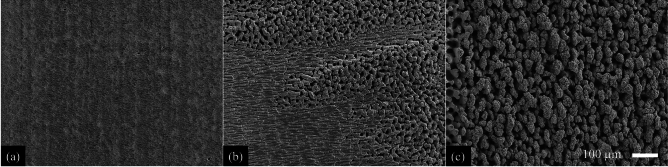
Resulting topography after irradiation of the steel samples using single pulses with a fluence of **a** 50 J/cm$^2$, **b** 75 J/cm$^2$ and **c** 200 J/cm$^2$ after one scan.

**Fig. 3:**
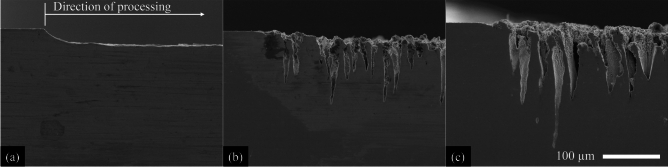
Cross sections of the irradiated steel samples using single pulses with a fluence of **a** 50 J/cm$^2$, **b** 75 J/cm$^2$ and **c** 200 J/cm$^2$ after one scan.

In general, this study assumes that the formation of deep hole-like structures or CLPs at lower measurement angles relative to the material surface leads to a reduction in the measured X-ray photons due to absorption within the material. In the single-pulse regime, no angular dependence of X-ray emission was observed at single-pulse fluences of 25 J/cm$^2$ and 50 J/cm$^2$, as both the measured photon count and the spectral distribution remained nearly constant across all detection angles (see Fig. 1 for 50 J/cm$^2$). The surface morphology remained homogeneous (Fig. 2) and did not exhibit periodic microstructures or holes that could have caused angle-dependent variations in X-ray emission detection (Fig. 3a). Doubling the single-pulse fluence from 25 J/cm$^2$ to 50 J/cm$^2$ resulted in a significant increase in the detected photon count, from approximately 2200 to 12,200 photons, while the volume removed per pulse, $V_1$, only slightly increased from 127.3 $\upmu$m$^3$ to 138.1 $\upmu$m$^3$ (Table 1). When using ultrashort laser pulses, the laser-induced plasma evolves following the laser-material interaction^34–37^, with its expansion, electron dynamics, and ionization degree significantly affecting bremsstrahlung and characteristic X-ray emission^38–40^.

Using a fluence of 75 J/cm$^2$, the highest photon count of 1,860,000 counts was measured at the steepest investigated detection angle of 75$^{\circ }$ relative to the material surface (Fig. 1). Compared to a single-pulse fluence of 50 J/cm$^2$, the number of detected photons increased by a factor of 130. This strong increase is associated not only with the increase in single-pulse fluence but also with the formation of cauliflower-like structures and CLPs (Figs. 2b and 3b), which enhance laser absorption through multiple reflections and contribute to increased X-ray emission up to a maximum detected photon energy of 42 keV (Fig. 1). A pronounced angular dependence of the detected photon count first appeared at 75 J/cm$^2$, showing a continuous decrease in photon count with decreasing detection angles. At 15$^{\circ }$, only 38 % of the photons detected at 75$^{\circ }$ were measured. This shielding effect can be attributed to the absorption of X-ray radiation within the CLPs, whereby primarily radiation emerging nearly perpendicular to the material surface reaches the detector without being absorbed by the surrounding material (Fig. 3). Using fluences of 100 J/cm$^2$ and above, the measured photon count decreased at all investigated detection angles compared to a single-pulse fluence of 75 J/cm$^2$. The formation of deeper CLPs led to increased shielding effects, which became noticeable even at a detection angle of 75$^{\circ }$ (Fig. 3). As the fluence increased, the shielding effect became more pronounced: At 100 J/cm$^2$, only 30 % of the photons at 15$^{\circ }$ were retained compared to those at 75$^{\circ }$. At 150 J/cm$^2$, this ratio dropped to 21 %, and at 200 J/cm$^2$ only 16 % of the original photon count was detected.

In summary, the surface morphology generated by single-pulse ablation significantly influences the emitted photon flux density within the investigated parameter range. The formation of cauliflower-like structures and CLPs leads to a substantial increase in the detected photon count, which reaches its maximum at 75 J/cm$^2$. However, as these structures deepen, they increasingly shield X-ray radiation, particularly at lower detection angles. Since no significant periodic microstructures formed at single-pulse fluences at 25 and 50 J/cm$^2$, the X-ray photon count remained independent of the detection angle in this single-pulse fluence range, serving as a reference for the subsequent comparison of the emitted photon count per pulse in the following sections.

**Table 1:** Ablated volume $V_1$ per pulse as a function of the fluence per pulse using single pulse regime.

	25 J/cm$^2$	50 J/cm$^2$	75 J/cm$^2$	100 J/cm$^2$	150 J/cm$^2$	200 J/cm$^2$
$V_1$ ($\upmu$m$^3$)	127.3±5.6	138.1±13.4	195.1±20.2	217.2±33.7	275.7±40.6	375.1±42.2

### MHz-burst pulse ablation

Analogous to single-pulse ablation at a single-pulse fluence of 25 J/cm$^2$ and 50 J/cm$^2$, the analysis of the resulting surface morphology at a burst fluence of 50 J/cm² and 100 J/cm² reveals a largely homogeneous surface structure (Fig. 6a and b) without the formation of CLPs or similar hole-like microstructures (Fig. 7a and b). Minor differences in the resulting surface morphology compared to single-pulse ablation can be attributed to redeposition of ablated particles^41^, recoil pressure effects^42^, and heat accumulation^43^. Given the comparable surface morphology observed in the single-pulse regime up to 50 J/cm$^2$ and in MHz-burst pulses up to a burst fluence of 100 J/cm² consisting of two burst pulses with 50 J/cm$^2$ each, conclusions can also be drawn regarding the number of emitted photons per pulse.

The plasma evolution following the interaction of the first pulse with the material surface and the associated X-ray emission is completed within a few nanoseconds^34^. Due to the intra-burst delay of 15.4 ns, the contribution of the second pulse can be determined by calculating the difference between the total number of detected photons and the photon count from the first pulse. Subsequent comparisons are made at the maximum investigated measurement angle of 75$^{\circ }$ relative to the material surface.

**Fig. 4:**
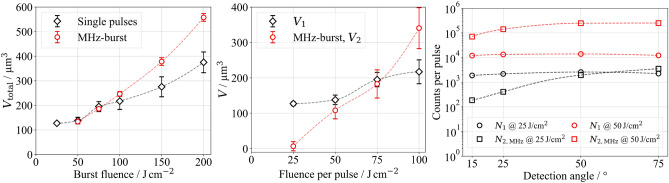
Ablated volume $V_\text {total}$ per MHz-burst as a function of burst fluence, including reference data from the single-pulse regime (left). Ablated volumes $V_1$ and $V_2$ per pulse (middle) represent the first and second pulse, respectively. $V_2$ is calculated using Eq. (2) as the difference between total and first-pulse volume. Negative values of $V_2$. Measured photon counts per pulse (right) with the first pulse $N_1$ and the second pulse $N_2$ as a function of the fluence per pulse and the detection angle.

**Fig. 5:**
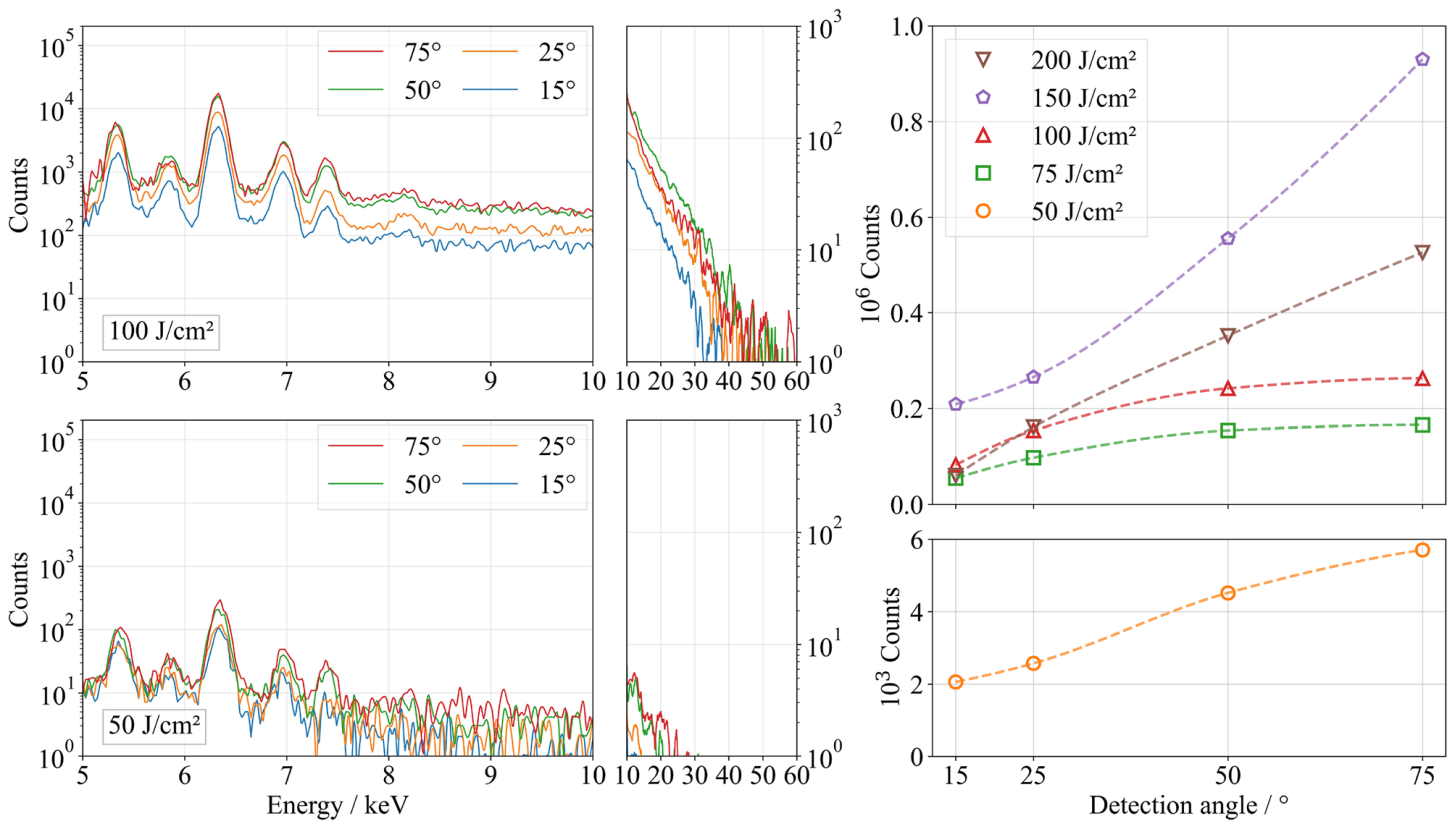
X-ray emission spectra (left) and the number of photons measured (right) as a function of the detection angle and the burst fluence using MHz-burst pulses.

Using a burst fluence of 50 J/cm$^2$, the ablated volume per burst, at 131 $\upmu$m$^3$, is comparable to the value measured in the single-pulse regime at a single-pulse fluence of 25 J/cm$^2$ (Fig. 4, left). Consequently, the second pulse in the MHz-burst configuration does not contribute to material ablation, as evident from the ablated volume per pulse (Fig. 4, middle). In the study by Förster et al., the absorption of the second pulse’s energy at a temporal pulse spacing of 12.5 ns led to the formation of a secondary plasma at the upper interface or within the ablation plume^41^, a phenomenon also observed in this study based on the emitted X-ray photons per pulse. At a single-pulse fluence of 25 J/cm$^2$, 2200 photons were detected, while an additional 3500 counts were recorded for the second pulse within a MHz burst (Fig. 4, right). Furthermore, with an applied burst fluence of 50 J/cm$^2$, the maximum detected photon energy increased slightly from 20 keV (Fig. 1) in the single-pulse regime at a fluence of 50 J/cm$^2$ to 25 keV in MHz-burst pulses (Fig. 5).

Increasing the fluence per burst pulse to 50 J/cm$^2$, corresponding to a burst fluence of 100 J/cm$^2$, results in a significant increase in the detected photon count at the same measurement angle: 12,200 photons for the first pulse and 250,000 for the second pulse (Fig. 4, right). Additionally, the maximum detected photon energy rises to 60 keV (Fig. 5). This significant increase in emitted photon count and photon energy can also be attributed to the interaction of the second pulse with the existing ablation plume, generating a markedly more intense secondary plasma at the upper interface or within the ablation plume.

In contrast to single-pulse ablation, a pronounced angular dependence of the detected photon count is already evident at a burst fluence of 50 J/cm$^2$ (Figs. 4 and 5). The photon count decreases from 5700 at 75$^{\circ }$ to 2100 at 15$^{\circ }$, despite the absence of significant microstructures in the surface morphology that could cause shielding effects. The observed angular dependence can be explained by the absorption of emitted X-ray radiation within the ablation plume. Since this plume expands over several micrometers^41,44^ within 15.4 ns, it acts as a transient medium for X-ray radiation.

**Fig. 6:**
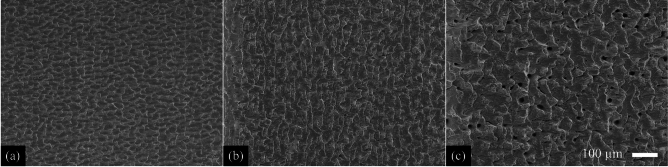
Resulting topography after irradiation of the steel samples using MHz-burst pulses with a burst fluence of **a** 50 J/cm$^2$, **b** 100 J/cm$^2$ and **c** 200 J/cm$^2$ after one scan.

**Fig. 7:**
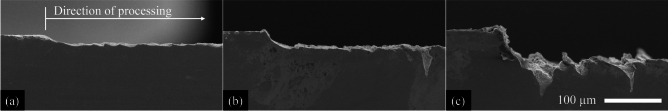
Cross sections of the irradiated steel samples using MHz-burst pulses with a burst fluence of **a** 50 J/cm$^2$, **b** 100 J/cm$^2$ and **c** 200 J/cm$^2$ after one scan.

The highest total detected photon count of 930,000 was measured at a burst fluence of 150 J/cm$^2$ (Fig. 5). Nevertheless, this total photon yield remains only half as high as the maximum achieved in the single-pulse mode at a single-pulse fluence of 75 J/cm$^2$. At a burst fluence of 200 J/cm$^2$, the total photon count decreases again, which may be related to the formation of holes in the surface morphology (see Figs. 6c and 7c). The observed effects resemble those of deeper CLPs in the single-pulse mode. In particular, in a burst configuration with two pulses of 100 J/cm$^2$ each, the second pulse removes significantly more material (340 $\upmu$m$^3$) than the first pulse (Fig. 4, right).

A possible explanation for this is that the recoil pressure of redeposited particles enhances the ejection of molten material from the surface, as described by Park et al.^33^. The X-ray radiation generated by the second pulse could then be partially absorbed within this ejected molten material, contributing to the observed decrease in detected photon count.

The degree of shielding, characterized by the reduction in photon count from 75$^{\circ }$ to 15$^{\circ }$, is nearly identical to that observed in the single-pulse mode. However, in the MHz-burst regime, additional factors must be considered, particularly the interaction of the second pulse with the existing ablation plume. While this interaction enhances X-ray emission, it can also introduce additional shielding effects due to ablated particles. Furthermore, angle-dependent melt ejection, caused by the recoil pressure of redeposited particles, may lead to additional absorption of X-ray radiation within the ejected melt droplets, further influencing the angle-dependent detection of photon counts.

### GHz-burst pulse ablation

Using GHz-burst pulses, no angular dependence of detected photon counts or X-ray spectra was observed throughout the investigated fluence range (Fig. 9). This can be attributed to the nearly unchanged surface morphology and minimal topographical modification during processing (Fig. 10). Consequently, subsequent bursts interact with an almost identical surface, preventing the formation of structures that could lead to angle-dependent detection of X-ray radiation (Fig. 11). Analogous to the findings of MHz-burst pulse ablation, conclusions regarding the number of emitted photons per pulse can be drawn based on the comparable surface morphology from the single-pulse regime up to a single-pulse fluence of 50 J/cm$^2$ and in GHz-burst pulses up to a burst fluence of 100 J/cm$^2$.

A key difference between MHz- and GHz-burst pulses is the temporal pulse separation within a burst, which is 15.4 ns in the MHz-burst regime and 400 ps in the GHz-burst regime. Semerok et al.^37^ demonstrated in double-pulse experiments that at a fluence per pulse of 30 J/cm$^2$ and within a pulse duration range of 50 fs to 2 ps, maximum plasma reheating occurs at the longest investigated delay time of 235 ps. Thus, the plasma generated by the first pulse, especially at higher fluences as examined in this study, could still persist within a GHz-burst pulse repetition time of 400 ps. Consequently, the second pulse in a GHz-burst may still interact with an existing plasma, making an exact differentiation of the detected X-ray photons challenging.

**Fig. 8:**
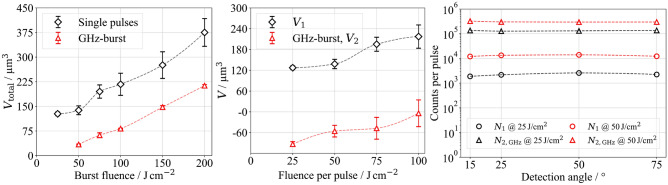
Ablated volume $V_\text {total}$ per GHz-burst as a function of burst fluence, including reference data from the single-pulse regime (left). Ablated volumes $V_1$ and $V_2$ per pulse (middle) represent the first and second pulse, respectively. $V_2$ is calculated using Eq. (2) as the difference between total and first-pulse volume. Negative values of $V_2$. Measured photon counts per pulse (right) with the first pulse $N_1$ and the second pulse $N_2$ as a function of the fluence per pulse and the detection angle.

**Fig. 9:**
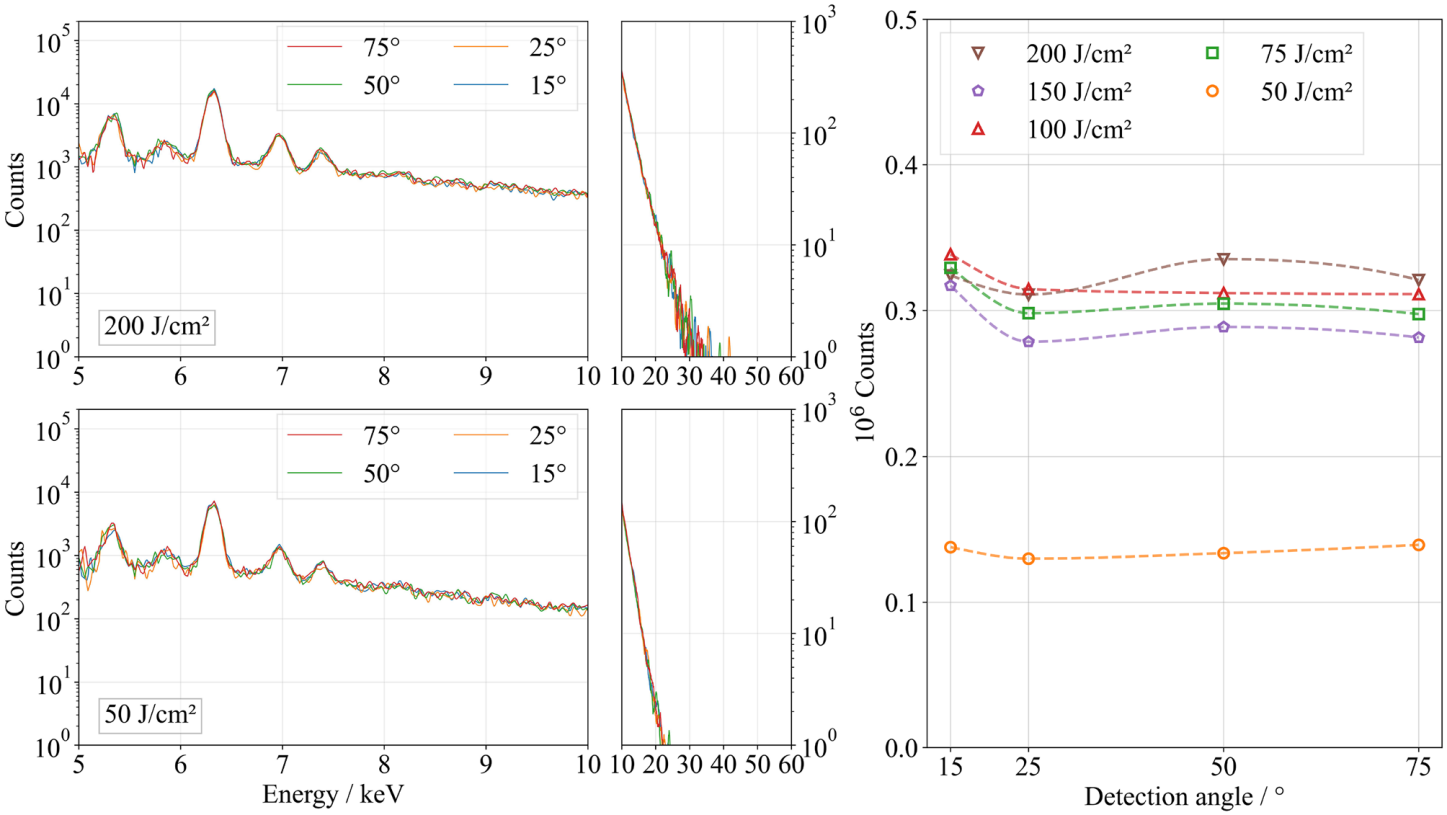
X-ray emission spectra (left) and the number of photons measured (right) as a function of the detection angle and the burst fluence using GHz-burst pulses.

Using a single-pulse fluence of 25 J/cm$^2$, 2200 counts were detected (Fig. 1), while 137,000 photons were detected with the second GHz pulse (Fig. 8, right). In comparison, only 3500 counts were detected for the second MHz-burst pulse at an identical burst fluence (Fig. 5, right). This clearly illustrates that pumping an existing plasma significantly increases the total number of emitted X-ray photons, compared to generating a second plasma, as occurs in the MHz-burst regime. Analysis of the volume ablated per pulse further reveals that at a burst fluence of 50 J/cm$^2$, the second pulse within the GHz-burst primarily contributes to the redeposition of the material ablated by the first pulse (Fig. 8, middle). The spectral distribution of the emitted X-ray radiation also remains comparable to that observed in the single-pulse regime (Fig. 9 compared to Fig. 1). While the second pulse increases the total photon count, it does not lead to a shift in the spectral energy distribution toward higher photon energies. This differs from the behavior in the MHz-burst regime, where such a shift was clearly observed (Fig. 5). Using a burst fluence of 100 J/cm², 12,200 X-ray photons were detected in the single-pulse regime at a single-pulse fluence of 50 J/cm$^2$ (Fig. 1). The second GHz-burst pulse 320,000 X-ray photons were detected after differentiation (Fig. 8, right).

Beyond a burst fluence of 75 J/cm$^2$, saturation of the detected photon count occurs at approximately 320,000 counts, which persists up to the highest investigated total fluence of 200 J/cm$^2$ (Fig. 9). Interestingly, this saturation is not reflected in the total ablated volume $V_{\text {total}}$, which continues to increase with higher fluence (Fig. 8, left).

**Fig. 10:**
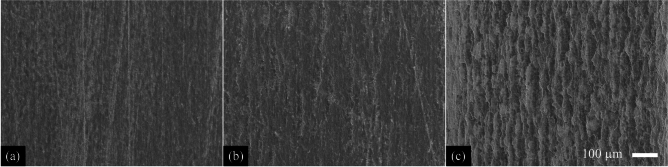
Resulting topography after irradiation of the steel samples using GHz-burst pulses with a burst fluence of **a** 50 J/cm$^2$, **b** 100 J/cm$^2$ and **c** 200 J/cm$^2$ after one scan.

**Fig. 11:**
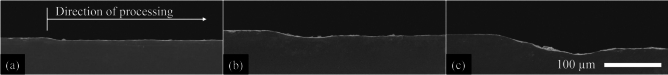
Cross sections of the irradiated steel samples using GHz-burst pulses with a burst fluence of **a** 50 J/cm$^2$, **b** 100 J/cm$^2$ and **c** 200 J/cm$^2$ after one scan.

This suggests that the saturation of the photon count is not directly related to the amount of ablated material.To clarify this divergence, Fig.8 (middle) distinguishes between the ablated volumes of the first and second pulse in a GHz-burst. The second-pulse volume $V_2$ is calculated using Eq. (2) as the difference between the total ablated volume and the contribution of the first pulse. Negative values of $V_2$, as observed in the GHz-burst regime, indicate redeposition of material ablated by the first pulse rather than additional removal. This redeposition may result from plasma-plume interaction or residual energy coupling of the second pulse into an overdense plasma, where further hot-electron generation is suppressed. In comparison, with the second MHz-burst pulse at this fluence, 250,000 photons were detected. Thus, the observations made for a burst fluence of 50 J/cm$^2$ also apply to 100 J/cm$^2$. This behavior is consistent with the interaction of the second pulse with an overdense plasma generated by the first pulse, where hot-electron generation and subsequent bremsstrahlung emission are limited due to partial laser reflection at the critical density boundary^45–49^. The electron density of the primary plasma is influenced by several factors, including the intensity of the first pulse, the temporal evolution of the plasma before interacting with the second pulse, and the intensity of the second pulse itself. As described by Barkauskas et al.^19^, the energy distribution within the burst plays a crucial role in determining the photon flux density during GHz-burst pulse ablation.

**Fig. 12:**
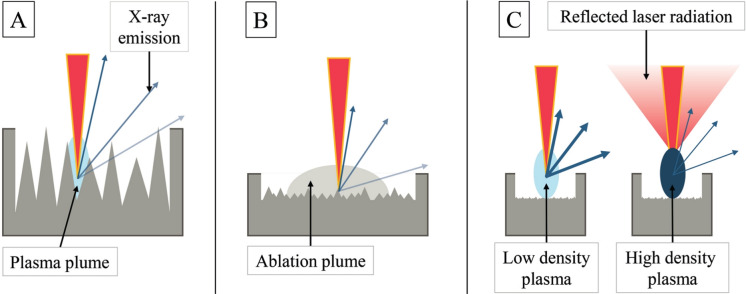
Schematic representation of the dominant mechanisms affecting X-ray emission: **A** Shielding due to surface topography in single-pulse ablation; **B** Shielding by the ablation plume in MHz-burst ablation; **C** Plasma-induced saturation effects in GHz-burst ablation, including partial reflection of the second pulse. Red cones represent laser pulses, blue ellipses indicate plasma regions, and blue arrows show X-ray emission. Reduced opacity of selected arrows qualitatively represents angular shielding effects.

To complement the detailed findings above, Fig. 12 schematically summarizes the primary mechanisms influencing the angular distribution and intensity of X-ray emission across the three irradiation regimes investigated. In the single-pulse regime (Fig. 12A), deep structures formed by repeated ablation can cause geometrical self-shielding of emitted X-rays, especially at lower angles. For MHz-burst irradiation (Fig. 12B), the second pulse interacts with an ablation plume generated by the first pulse. Despite a relatively smooth surface, the lateral expansion of the plume can lead to angular shielding of emitted X-rays. Figure 12C depicts two plasma conditions for the second pulse in the GHz-burst regime: in the left schematic, moderate plasma density allows transmission and further heating, resulting in enhanced X-ray emission; in the right schematic, a dense plasma reflects part of the second pulse, suppressing hot-electron generation and leading to saturation of the X-ray yield. These schematics highlight how plume shielding, plasma density, and surface morphology combine to influence emission characteristics.

## Discussion

The results of this study reveal a strong correlation between surface morphology, plasma dynamics, and the angular dependence of detected X-ray emission during ultrashort pulse laser ablation in single and burst pulse regimes. The evolution of the X-ray emission characteristics strongly depends on the pulse energy, the temporal pulse separation within bursts, and the resulting plasma interactions.

In the single-pulse regime, angular dependence of the X-ray emission was observed only at single-pulse fluences of 75 J/cm$^2$ and above. This behavior is directly linked to the formation of characteristic surface structures, such as cone-like protrusions (CLPs) and cauliflower-like morphologies, which significantly enhance laser absorption through multiple reflections. At high fluences, these structures increasingly shield X-rays emitted at lower detection angles due to geometric absorption effects within the surface topography. Below this fluence threshold, the surface morphology remains largely homogeneous, and X-ray emission exhibits no significant angular dependence.

In contrast, MHz-burst pulses induce angular-dependent X-ray emission even at lower burst fluences. Despite surface morphologies comparable to the single-pulse regime, a pronounced angular dependence emerges due to interactions between subsequent pulses within a burst and the ablation plume. This interaction leads to enhanced secondary X-ray emission but also introduces additional shielding effects. Furthermore, redeposition effects, recoil pressure, and melt dynamics influence the surface morphology, contributing to altered absorption and scattering of X-rays.

In the GHz-burst regime, no angular dependence of X-ray emission was detected across the entire investigated burst fluence range. The consistently homogeneous surface morphology and the absence of pronounced microstructuring prevent topography-induced shielding effects. A key differentiating factor compared to the MHz-burst regime is the interaction between the second pulse within the burst and the existing plasma. Due to the extremely short pulse separation of 400 ps, the second pulse interacts with a still-existing plasma, leading to a significant increase in total X-ray emission. However, despite increasing ablation volumes at higher fluences, the total photon count saturates beyond a burst fluence of 75 J/cm². This suggests a decoupling of X-ray yield from the ablation volume, which can be attributed to plasma shielding. When the electron density within the plasma exceeds a critical threshold, a portion of the second pulse’s energy is reflected or absorbed at the plasma boundary, limiting further X-ray generation.

On the basis of these findings, future investigations should focus on the transient dynamics of plasma formation and its interaction with subsequent pulses in burst regimes. In particular, the role of the ablation plume in MHz-burst ablation and potential plasma shielding effects in GHz-burst ablation require further clarification. Special attention should be given to the energy distribution within the burst and the temporal separation between pulses, as these parameters are expected to significantly influence both plasma behavior and X-ray emission. Time-resolved and spatially resolved diagnostics will be essential to deepen the understanding of these processes and to optimize burst strategies for controlled X-ray generation.

## Methods

### Optical setup

An amplified solid-state laser system (Carbide CB3-80, Light Conversion) was utilized for the experiments, operating at a wavelength ($\lambda$) of 1030 nm with an average power ($P_{\text {av}}$) of up to 80 W, a pulse repetition rate (*f*) ranging from single shot to 2 MHz and a tunable pulse duration ($\tau _{\text {H}}$) between 200 fs and 10 ps. The spatial intensity profile of the beam closely resembled an ideal Gaussian distribution ($M^2$ $\le$ 1.13). The laser was capable of generating burst pulses with inter-burst pulse repetition rates of 65 MHz and 2.5 GHz, corresponding to time delays of approximately 15.4 ns and 400 ps between consecutive burst pulses, respectively. The burst repetition rate equalled those of the laser source in the non-burst regime. A spherical f($\Theta$) lens with a focal length of 163 mm focused the laser radiation onto the material surface, achieving a beam waist radius ($w_0$) of 13.3 $\upmu$m, determined using a Primes MicroSpot-Monitor according to ISO 11146^50^. To guide the laser beam across the sample surface, a galvanometer scanner system was employed.

### Material and process parameters

Rectangular plane areas with an edge length of 25 mm were irradiated on 1 mm thick cold-rolled steel, characterized as a standard AISI 304L-steel grade containing primarily Fe, Cr and Ni. All experiments were conducted in air at atmospheric pressure.

To maximize the available pulse energy, both burst and single-pulse modes were operated at the laser’s lowest internal repetition frequency of 100 kHz. However, to prevent subsequent bursts from being influenced by the material removed during the previous burst^51–53^, a pulse picker with a factor of 10 was used, effectively reducing the burst repetition frequency to 10 kHz. The laser radiation was deflected across the sample surface with a geometric burst hatch distance *dx* of 3 $\upmu$m, corresponding to a scanning speed of 0.03 m/s. Multiple lines with a line hatch distance *dy* of 3 $\upmu$m were used to realize an area irradiation of (25 x 1) mm$^2$ comparable to a laser scanning milling process with a single area scan. Consequently, a area irradiation for each parameter results in a processing time of 283 s.

To achieve maximum peak power and intensity, the shortest available pulse duration of 200 fs was selected for all parameters in this study. In consideration of parasitic effects related to intensity limitations, critical thresholds were carefully taken into account. In the single-pulse regime, a pulse energy range of 70 $\upmu$J to 555 $\upmu$J was investigated, corresponding to a single-pulse fluence range of 25 J/cm$^2$ to 200 J/cm$^2$. This results in a maximum peak power of 2.8 GW, which remains below the critical peak power of 5 GW at a wavelength of 1030 nm, where parasitic effects such as self-focusing or intensity-limiting filamentation can occur^54,55^. With the 200 fs pulse duration, the resulting intensity range in the single-pulse regime was calculated to be 3$\cdot$10$^{13}$ W/cm$^2$ to 9$\cdot$10$^{15}$ W/cm$^2$.

To enable a meaningful comparison of X-ray emission between single and burst pulses, two essential aspects were considered:

- Parameter ranges were selected so that the burst fluence and a single-pulse fluence was identical.
- The energy distribution within a burst was chosen to ensure comparable fluence per burst pulse across the two investigated pulses in a burst (Figure S.1). This methodology allows for a direct comparison between single pulses and individual pulses within a burst. For example, a single-pulse fluence of 50 J/cm$^2$ was evaluated using single pulses and double pulses per burst, with each pulse contributing 50 J/cm$^2$. As a result, the interaction of the first pulse in a burst with the material, and its associated X-ray emission, is directly comparable to that of a single pulse with the same fluence. Furthermore, the influence of the second burst pulse on the resulting X-ray emission and surface morphology can be analyzed independently.

### Surface morphology analysis

Confocal laser scanning microscopy (Olympus Lext 3D OLS4100) was conducted for quantitative analysis of the resulting ablation depth (*z*), performed according to ISO 25178^56^. In addition, qualitative analyses were performed using scanning electron microscopy (SEM, Jeol JSM-6512 V).

To characterize the depth profiles of the resulting topographies after irradiation, the samples were embedded in resin (Technovit 5071) for cross-sectional preparation. After curing, the resin-embedded samples were ground and polished in three sequential steps. Initial grinding was performed with abrasive paper (SiC 320). This was followed by fine grinding using a diamond suspension (monocrystalline diamond, 9 $\upmu$m and 3 $\upmu$m, Struers GmbH Dia-Duo) and completed with final polishing using an oxide polishing suspension (SiO$_2$, 0.04 $\upmu$m, Struers GmbH OP-S NonDry).

The ablated volume per pulse provides insights into mechanisms such as particle shielding, plasma shielding, and re-deposition of ablated material within a burst. This analysis is motivated by the potential interactions between subsequent burst pulses and ablated particles or existing plasma, which may influence both the emission of X-rays and the resulting surface morphology.

The ablated volume per pulse ($V_1$) is determined using the following equation^57^:

1 $$\begin{aligned} V_{\text {1}}=\Bigg [\frac{dx\;dy\,z}{n_{\text {scan}}}\Bigg ]\,\upmu \text {m}^3. \end{aligned}$$

Here, *dx* and *dy* represent the geometric burst and line hatch distances, *z* is the measured ablation depth of the irradiated material, and $n_{\text {scan}}$ denotes the number of scans, which is set to 1 in this study. The ablated volume of the second pulse within a burst ($V_2$) is calculated as the difference between the total ablated volume ($V_{\text {total}}$) using double pulses and the ablated volume of the first pulse ($V_1$) at comparable fluence per pulse:

2 $$\begin{aligned} V_{\text {2}}=\Bigg [V_{\text {total}}-V_1\Bigg ]\,\upmu \text {m}^3. \end{aligned}$$

Note that $V_1$ and $V_2$ are calculated at identical fluence per pulse (e.g., 50 J/cm$^2$), while $V_{\text {total}}$ corresponds to the combined burst fluence (e.g., 2 $\times$ 50 J/cm$^2$). This ensures that $V_2 = V_{\text {total}} - V_1$ accurately reflects the contribution of the second pulse within a double-pulse burst at matched individual pulse energies. This calculation methodology is consistently applied throughout the study to analyze pulse-specific ablation behavior across different burst regimes.

### X-ray analysis

#### Measurement device

Time-resolved measurements of laser-induced X-ray emission spectra were performed using a silicon drift detector (SDD) spectrometer in single-photon counting mode with a channel width of 30 eV. The fundamental setup of the X-ray spectrometer consists of incident photons passing through a 25 $\upmu$m thick Be protective shield and a collimator before interacting with a thermoelectrically cooled Si-PN junction detector.

During X-ray emission measurements of plasmas induced by ultrashort laser pulses, two primary sources of signal distortion can occur. First, the high photon flux densities in the spectral range below 5 keV, and second, the pronounced characteristic emission lines of the material-specific elements, which may lead to detector saturation and photon-pile-up effects^58^. To mitigate these effects and prevent damage to the detector, Al foil filters of varying thicknesses were placed in front of the detector to attenuate the photon flux density.

#### Experimental setup

**Fig. 13:**
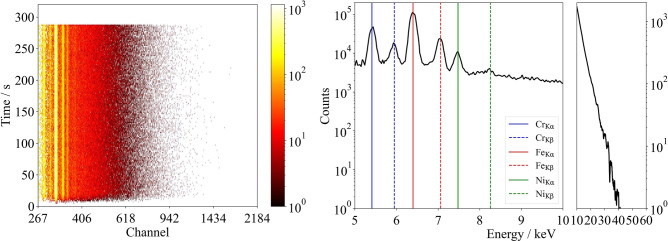
Left: Exemplary representation of the time-resolved X-ray emission spectra measurement. Right: Calibration procedure using the characteristic emission lines of Fe, Cr, and Ni.

The X-ray spectrometer was positioned at a fixed distance of 45 cm from the center of the interaction zone, with a variable detector angle relative to the material surface at 15$^{\circ }$, 25$^{\circ }$, 50$^{\circ }$, and 75$^{\circ }$. The direction of processing was bi-directional and oriented perpendicular to the detector. Each new scan line was generated with the predefined line hatch distance, moving away from the detector, to minimize potential shielding effects caused by the formation of flanks during the ablation process. The duration of measurement was defined to ensure that the detector was active shortly before and after the processing time of 283 s (Fig. 13, left). The conversion of counts per channel into counts per keV was performed using the characteristic emission lines of Fe, Cr, and Ni (Fig. 13, right). To enhance the clarity of the characteristic emission lines and the energy range up to 10 keV, the X-ray spectra were divided into two sections with different scaling factors. The range up to 10 keV was displayed with a broader scale to improve the visibility of variations in photon counts, while the range up to 60 keV was represented with a narrower scale, as this region was predominantly analyzed for maximum detected photon energies.

Additionally, a correction was applied to account for the absorption of the Al foil filters based on the known spectral absorption coefficient (NIST.com) and the quantum efficiency of the junction detector. Due to the lower photon flux density and quantum efficiency in the spectral energy range above 30 keV, the measured signal exhibits increased noise. To improve the clarity of the presented X-ray spectra, a Savitzky-Golay de-noising filter^59,60^ was applied for photon energies above 10 keV. However, it should be noted that the total number of detected photons per parameter was summed from the raw data, which are available in the supplementary materials.

## Supplementary Information

Supplementary Information.

## Data Availability

The datasets used and/or analysed during the current study are available from the corresponding author on reasonable request.
