# Supplementary material for: Influence of plasma, surface, and angle on interlinked X-ray emission dynamics in femtosecond burst pulse ablation
Source: Sci Rep. 2026 Jan 8;16:885. doi: 10.1038/s41598-025-34221-x (PMC12783818; doi:10.1038/s41598-025-34221-x)
Supplement: Supplementary file 1 — Supplementary Information. [file 41598_2025_34221_MOESM1_ESM.pdf]

Supplementary material to the study entitled "Influence of plasma, surface, and angle on interlinked X-ray emission dynamics in femtosecond burst pulse ablation"

Daniel Metzner[1,*], Philipp Rebentrost[1], Peter Lickschat[1], Thomas Lampke[2], and Steffen Weißmantel[1]

[1]University of Applied Sciences Mittweida, Laserinstitut Hochschule Mittweida, Mittweida, 09648, Germany
[2]Chemnitz University of Technology, Department of Materials and Surface Engineering, Chemnitz, 09125, Gwermany

ABSTRACT

The supplementary material contains additional information and raw data to facilitate the comprehension of the results presented.

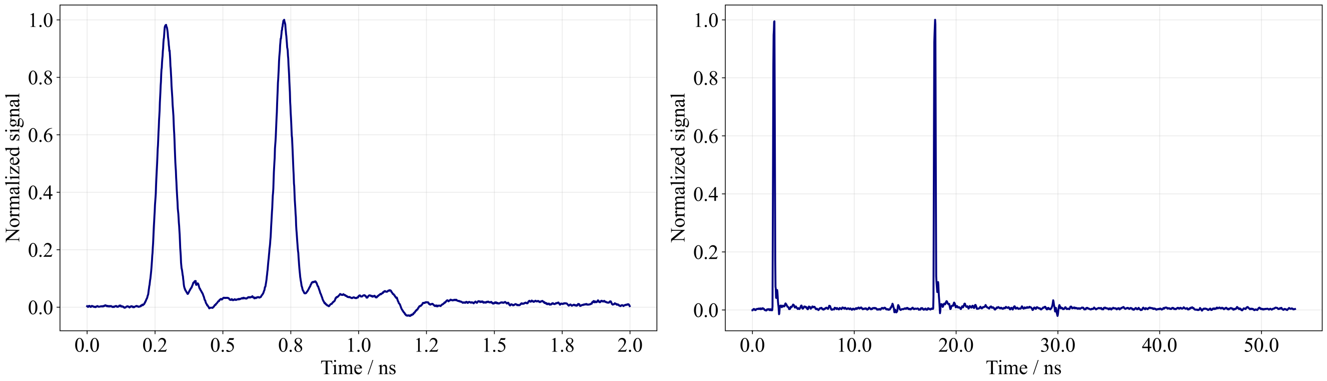

Figure S.1. Oscilloscope images of the energy distribution using GHz-burst pulses (left) and MHz-burst pulses (right).

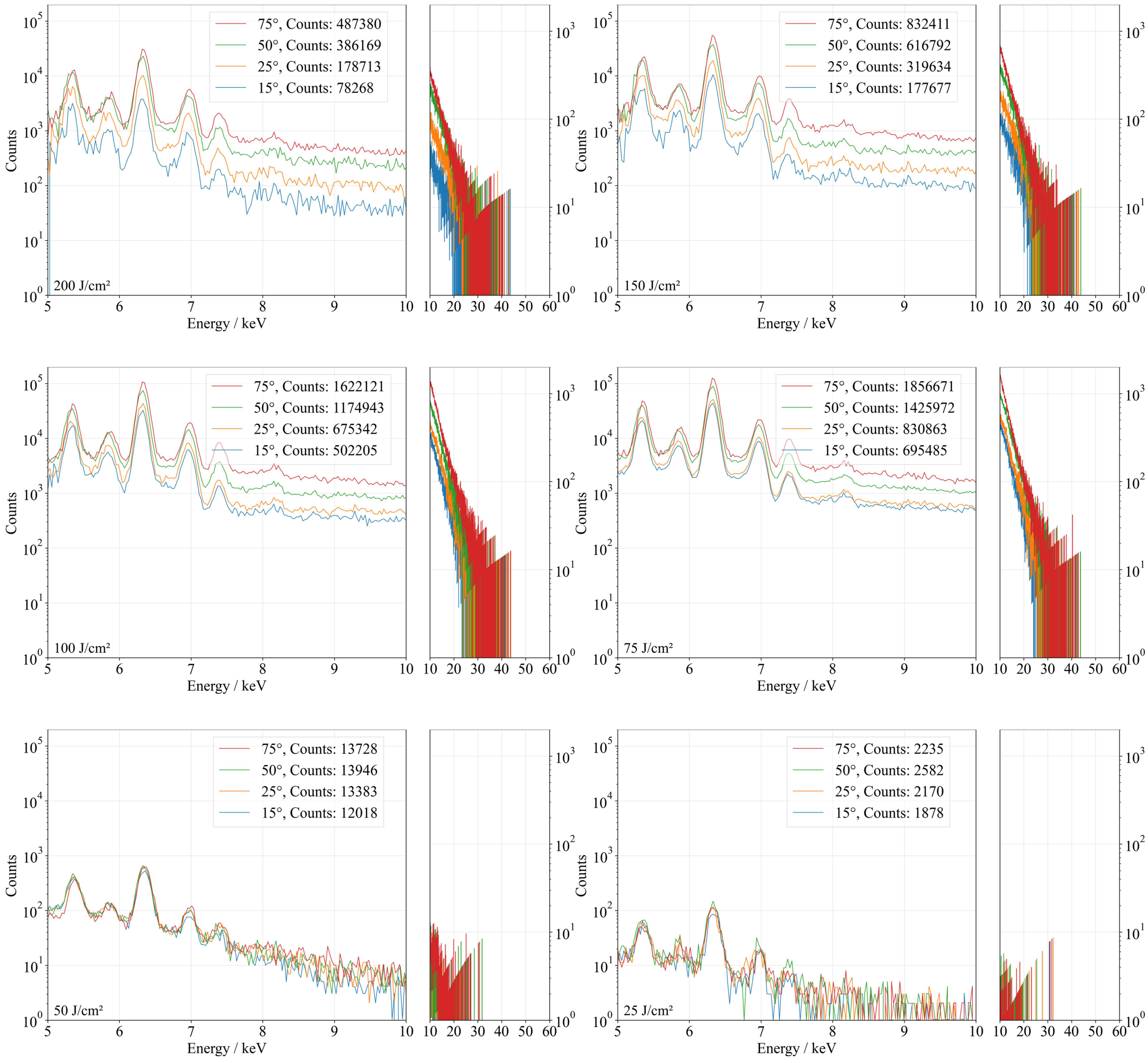

Figure S.2. X-ray emission spectra using single pulse regime with a single pulse fluence from 25 J/cm^2 up to 200 J/cm^2 at a function of the detection angle. The images including the total counts per parameter set.

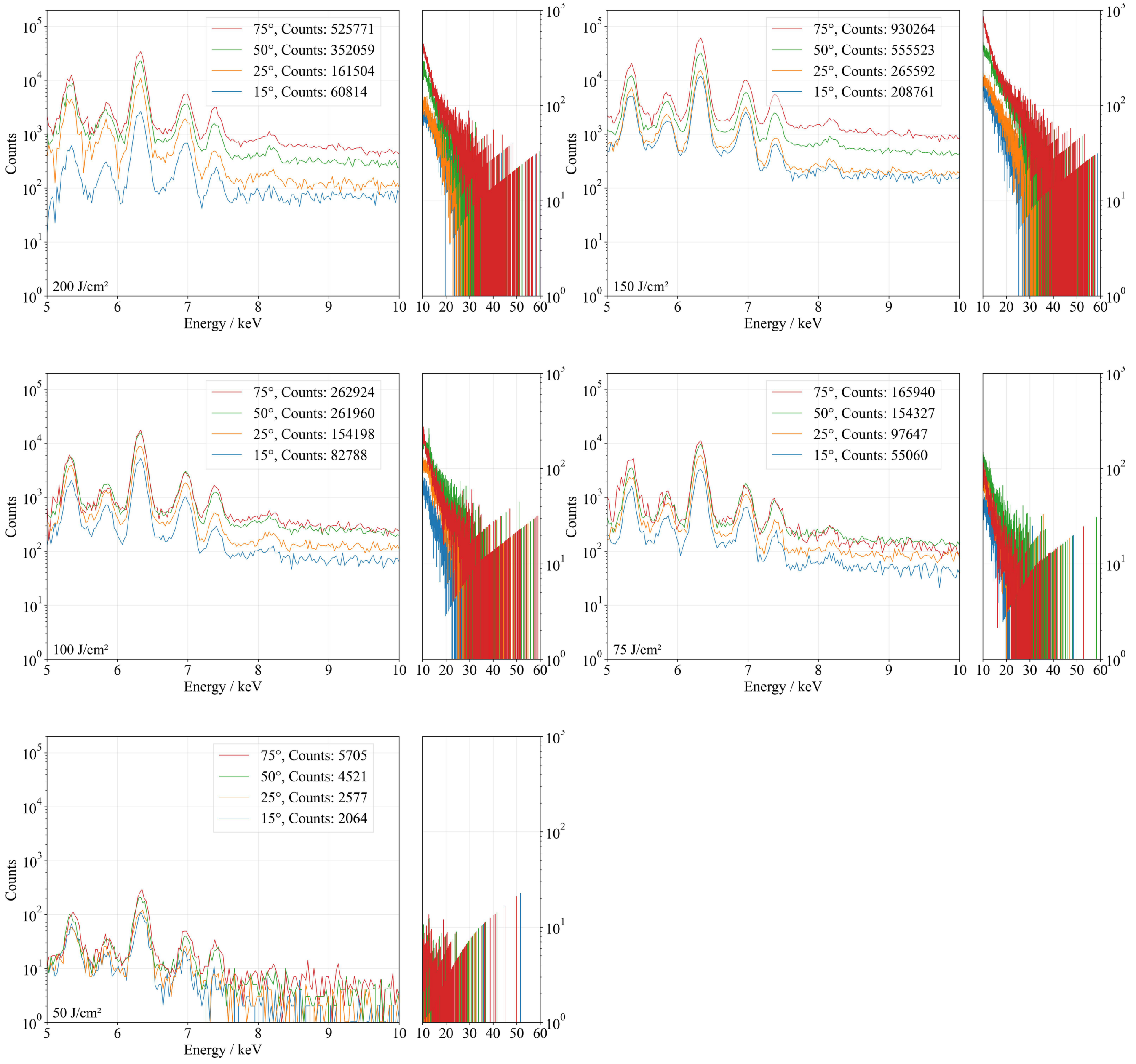

Figure S.3. X-ray emission spectra using MHz-burst pulses with a burst fluence from 50 J/cm^2 up to 200 J/cm^2 equally divided into two burst pulses at a function of the detection angle. The images including the total counts per parameter set.

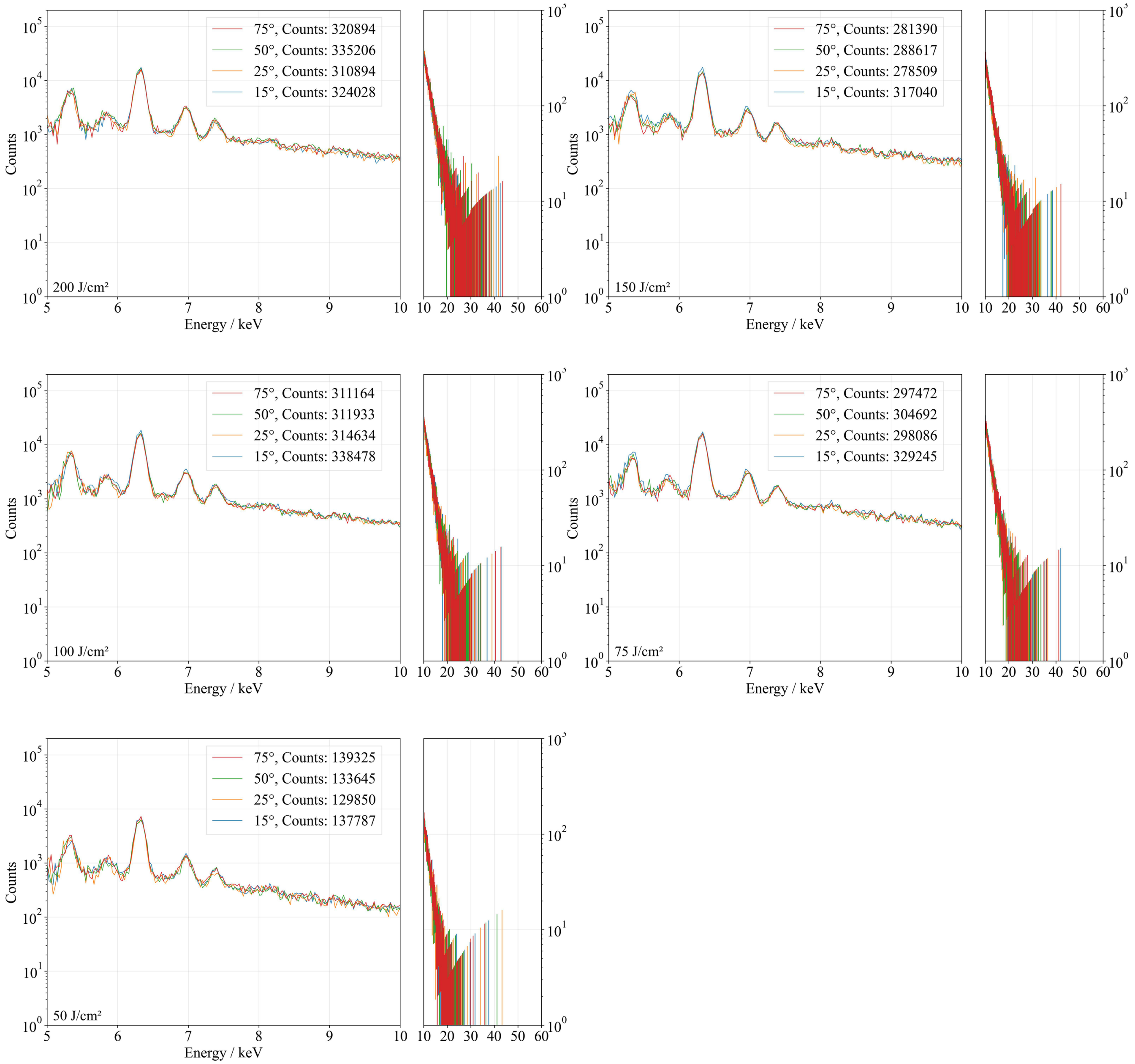

Figure S.4. X-ray emission spectra using GHz-burst pulses with a burst fluence from 50 J/cm^2 up to 200 J/cm^2 equally divided into two burst pulses at a function of the detection angle. The images including the total counts per parameter set.
